# Galcanezumab for migraine

**DOI:** 10.18773/austprescr.2020.044

**Published:** 2020-06-25

Approved indication: migraine

Emgality (Eli Lilly)

prefilled pen, prefilled syringe containing 120 mg/mL

Several drugs can be considered for prophylaxis in patients who have frequent migraines. The range of options was recently increased by the approval of erenumab, an injectable monoclonal antibody. Galcanezumab is another monoclonal antibody that acts on calcitonin gene-related peptide (CGRP).

Concentrations of CGRP increase during a migraine attack resulting in vasodilation. By binding to CGRP galcanezumab blocks this effect. The long half-life of galcanezumab (27 days) results in its action being sustained for several weeks.

A loading dose is recommended followed by monthly subcutaneous injections. As galcanezumab is an antibody it is catabolised, so hepatic and renal impairment are unlikely to affect its pharmacokinetics.

There have been two trials (EVOLVE 1 and EVOLVE 2) of galcanezumab in episodic migraine.^1^,^2^ The patients in these trials were experiencing 4–14 days of migraine headaches each month. After other prophylactic drugs were stopped, the patients were randomised to monthly injections of galcanezumab 120 mg or 240 mg, or placebo. Efficacy was assessed after six months.

In EVOLVE 1 (862 patients) the reduction in days of headache compared to placebo averaged 1.9 days with galcanezumab 120 mg and 1.8 days with galcanezumab 240 mg.^1^ The corresponding reductions in EVOLVE 2 (915 patients) were two days and 1.9 days (see Table).^2^

*Table* Efficacy of galcanezumab prophylaxis in episodic migraine

**Table:** 

Treatment (monthly subcutaneous injection)	Trial					
	Evolve 1 1			Evolve 2 2		
	Placebo	Galcanezumab 120 mg	Galcanezumab 240 mg	Placebo	Galcanezumab 120 mg	Galcanezumab 240 mg
Patients randomised	433	213	212	461	231	223
Mean number of migraine headache days per month at baseline	9.1	9.2	9.1	9.2	9.1	9.1
Mean reduction in migraine headache days per month after 6 months	2.8	4.7	4.6	2.3	4.3	4.2
Proportion of patients having at least a 50% reduction in migraine headache days per month at 6 months	38.6%	62.3%	60.9%	36%	59.3%	56.5%

The REGAIN trial studied patients with chronic migraine who had at least 15 days of headache every month. A group of 278 patients injected galcanezumab 120 mg, 277 injected 240 mg and 558 injected a placebo monthly. After three months, the number of days with migraine headache per month had reduced by a mean of 4.8 days with 120 mg and 4.6 days with 240 mg. These outcomes were statistically better than the reduction of 2.7 days seen in the placebo group. There was a reduction of at least 50% in the number of days with headache in 27.6% and 27.5% of the galcanezumab groups compared with 15.4% of the placebo group.^3^

A longer term open-label trial studied 270 patients with episodic or chronic migraine. Half the patients injected galcanezumab 120 mg and the other half injected 240 mg for up to a year. The mean number of days of migraine headache per month dropped by 5.6 days, from a baseline of 9.7, with 120 mg and by 6.5 days, from a baseline of 11.4, with 240 mg.^4^

In the long-term study 60 of the 270 patients stopped treatment. Eighteen discontinued because of lack of efficacy and 13 (4.8%) because of adverse events. Common adverse effects include reactions and pain at the injection sites, arthralgia, myalgia and dizziness. Injecting an immunoglobulin can cause immune reactions. After 12 months, 12.4% of the patients injecting the recommended monthly dose of 120 mg had developed antidrug antibodies.^4^ Anaphylaxis is rare. While there were few reports of cardiovascular effects in the trials, patients with a history of cardiovascular events were excluded. The safety of galcanezumab in pregnancy and lactation is also unknown. There are no paediatric data.

The place of drugs aimed at CGRP is currently being established. Galcanezumab is also being studied in cluster headache. While galcanezumab can reduce the number of days of migraine, it is uncertain whether it will work when other prophylaxis has failed. Patients whose migraine had not responded to three or more other drugs were excluded from the trials. If a patient tries galcanezumab, the response should be assessed after 8–12 weeks to see if it is worthwhile continuing to use it for prophylaxis.


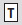
 manufacturer provided the product information

The Transparency Score is explained in New drugs: transparency, Vol 37 No 1, Aust Prescr 2014;37:27.

At the time the comment was prepared, information about this drug was available on the websites of the Food and Drug Administrationin the USA, and the European Medicines Agency.

## References

[1] Stauffer VL Dodick DW Zhang Q Carter JN Ailani J Conley RR Evaluation of galcanezumab for the prevention of episodic migraine: the EVOLVE-1 randomized clinical trial. JAMA Neurol 2018;75:1080-8. 10.1001/jamaneurol.2018.1212 29813147 PMC6143119

[2] Skljarevski V Matharu M Millen BA Ossipov MH Kim BK Yang JY Efficacy and safety of galcanezumab for the prevention of episodic migraine: Results of the EVOLVE-2 Phase 3 randomized controlled clinical trial. Cephalalgia 2018;38:1442-54. 10.1177/0333102418779543 29848108

[3] Detke HC Goadsby PJ Wang S Friedman DI Selzler KJ Aurora SK Galcanezumab in chronic migraine: The randomized, double-blind, placebo-controlled REGAIN study. Neurology 2018;91:e2211-21. 10.1212/WNL.0000000000006640 30446596 PMC6329331

[4] Camporeale A Kudrow D Sides R Wang S Van Dycke A Selzler KJ A phase 3, long-term, open-label safety study of Galcanezumab in patients with migraine. BMC Neurol 2018;18:188. 10.1186/s12883-018-1193-2 30413151 PMC6234796

